# Crystal structure of (2*E*)-1-(4-hy­droxy-3-meth­oxy­phen­yl)-3-(4-hy­droxy­phen­yl)prop-2-en-1-one

**DOI:** 10.1107/S1600536814021953

**Published:** 2014-10-15

**Authors:** S. Sathya, D. Reuben Jonathan, K. Prathebha, J. Jovita, G. Usha

**Affiliations:** a PG and Research Department of Physics, Queen Mary’s College, Chennai-4, Tamilnadu, India; b Department of Chemistry, Madras Christian College, Chennai-59, India

**Keywords:** crystal structure, prop-2-en-1-one, chalcones, biological activity, hydrogen bonding

## Abstract

In the title moleclue, C_16_H_14_O_4_, the dihedral angle between the benzene rings is 16.1 (3)°. The meth­oxy group is essentially coplanar with the benzene ring to which it is attached, with a C—O—C C torsion angle of 5.5 (9)°. In the crystal, mol­ecules are linked by O—H⋯O and bifurcated O—H⋯(O,O) hydrogen bonds, forming a three-dimensional network. The structure was refined as a two-component inversion twin.

## Related literature   

For the biological activity of chalcones, see: Prasad *et al.* (2008 ▶); Won *et al.* (2005 ▶); Yu *et al.* (1982 ▶); Ram *et al.* (2000 ▶); Khatib *et al.* (2005 ▶); Papo & Shai (2003 ▶). For related structures, see: Jasinski *et al.* (2011 ▶); Sathya *et al.* (2014 ▶). For the synthesis, see: Sidharthan *et al.* (2012 ▶); Chitra *et al.* (2013 ▶); Jasmine Francis *et al.* (2014 ▶).
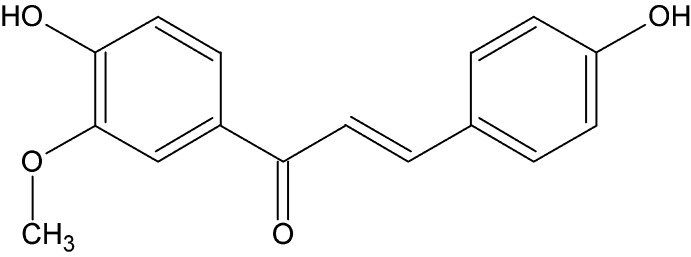


## Experimental   

### Crystal data  

C_16_H_14_O_4_
*M* *_r_* = 270.27
Orthorhombic, 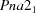

*a* = 7.686 (16) Å
*b* = 28.346 (7) Å
*c* = 6.297 (12) Å
*V* = 1371.9 (5) Å^3^
*Z* = 4
Mo *K*α radiation
μ = 0.09 mm^−1^
*T* = 293 K
0.35 × 0.30 × 0.25 mm

### Data collection  

Bruker Kappa APEXII diffractometer
Absorption correction: multi-scan (*SADABS*; Bruker, 2008 ▶) *T*_min_ = 0.968, *T*_max_ = 0.977
6436 measured reflections
2996 independent reflections
1928 reflections with *I* > 2σ(*I*)
*R*_int_ = 0.055
Standard reflections: ?

### Refinement  

*R*[*F* ^2^ > 2σ(*F* ^2^)] = 0.077
*wR*(*F* ^2^) = 0.244
*S* = 1.07
2996 reflections
186 parameters
1 restraint
H-atom parameters constrained
Δρ_max_ = 0.33 e Å^−3^
Δρ_min_ = −0.30 e Å^−3^
Absolute structure: refined as an inversion twin (1113 Friedel pairs)
Absolute structure parameter: −2 (4)

### 

Data collection: *APEX2* (Bruker, 2008 ▶); cell refinement: *SAINT* (Bruker, 2008 ▶); data reduction: *SAINT*; program(s) used to solve structure: *SHELXS2013* (Sheldrick, 2008 ▶); program(s) used to refine structure: *SHELXL2013* (Sheldrick, 2008 ▶); molecular graphics: *PLATON* (Spek, 2009 ▶); software used to prepare material for publication: *SHELXL2013* (Sheldrick, 2008 ▶).

## Supplementary Material

Crystal structure: contains datablock(s) I, New_Global_Publ_Block. DOI: 10.1107/S1600536814021953/lh5720sup1.cif

Structure factors: contains datablock(s) I. DOI: 10.1107/S1600536814021953/lh5720Isup2.hkl

Supporting information file. DOI: 10.1107/S1600536814021953/lh5720Isup3.cml

**Fig. 1.**
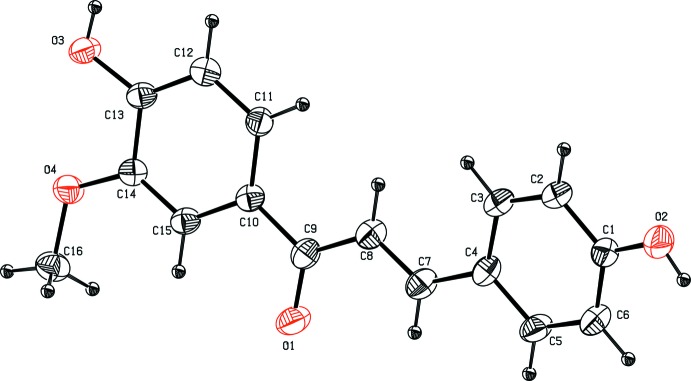
The molecular structure of the title compound, with displacement ellipsoids drawn at the 30% probability level.

. DOI: 10.1107/S1600536814021953/lh5720fig1.tif
The mol­ecular structure of the title compound, with displacement ellipsoids drawn at the 30% probability level.

**Fig. 2.**
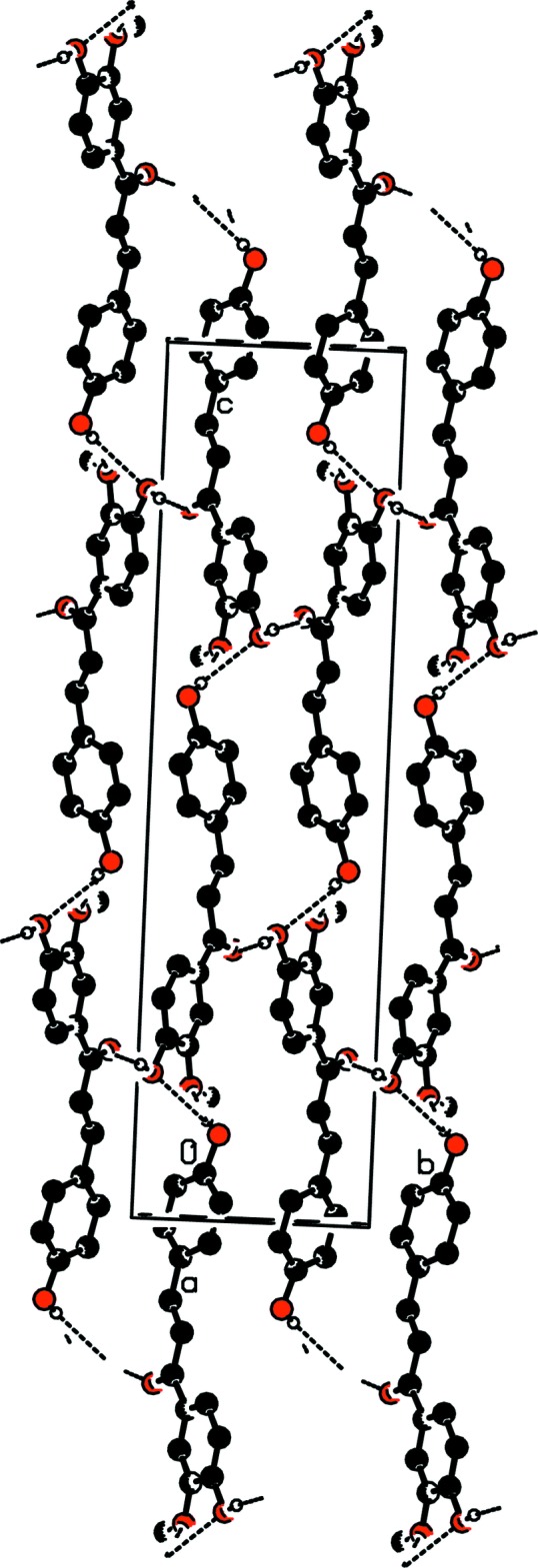
Part of the crystal structure with dashed lines indicating hydrogen bonds.

. DOI: 10.1107/S1600536814021953/lh5720fig2.tif
Part of the crystal structure with dashed lines indicating hydrogen bonds.

CCDC reference: 1011152

Additional supporting information:  crystallographic information; 3D view; checkCIF report

## Figures and Tables

**Table 1:** Hydrogen-bond geometry (, )

*D*H*A*	*D*H	H*A*	*D* *A*	*D*H*A*
O2H2*A*O3^i^	0.82	2.59	3.060(6)	118
O2H2*A*O4^i^	0.82	2.02	2.833(7)	172
O3H3*A*O1^ii^	0.82	1.87	2.618(7)	151

Symmetry codes: (i) 
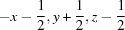
; (ii) 
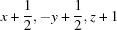
.

## supplementary crystallographic information

### S1. Comment

Chalcones constitute an important group of natural products due to their unforeseen pharmacological potential. Chemically, they consist of open chain flavanoids in which the two aromatic rings are joined by a three carbon alpha, beta unsaturated carbonyl system. The presence of a reactive alpha, beta unsaturated keto group in chalcones is mainly responsible for their antimicrobial activity (Prasad *et al.*, 2008). In recent years a variety of chalcones have been reviewed for their cytotoxic, anticancer chemopreventive and mutagenic as well as antiviral, insecticidal and enzyme inhibitory properties (Won *et al.*, 2005; Yu *et al.*, 1982). A number of chalcones having hydroxy, alkoxy groups in different position have been reported to possess vasodilatory (Ram *et al.*, 2000), antimitotic (Khatib *et al.*, 2005), antimalarial activities (Papo *et al.*, 2003). The enormous research potentials of these group of compounds motivated us to synthesize the title compound.

The molecular structure of the title compound is shown in Fig. 1. The bond lengths are comparable to literature values (Sathya *et al.*, 2014; Jasinski *et al.*, 2011). The C10—C9—C8 and C8—C7—C4 angles are slightly distorted compared to the values expected in terms of hybridization principles and this may be due to intra- and intermolecular steric interactions. In the crystal, molecules are linked by O—H···O and bifurcated O—H···(O,*O*) hydrogen bonds forming a three-dimensional network (Fig. 2 and Table 1).

### S2. Experimental

This acid catalyzed Claisen–Schmidt reaction and the procedure (Sidharthan *et al.*, 2012, Chitra *et al.*, 2013, Jasmine Francis*et al.*,2014) adopted in the synthesis of the typical chalcone diol namely (2E)-1-(4-hydroxy-3- methoxyphenyl)-3-(4-hydroxyphenyl)prop-2-en-1-one is represented herein. Dry HCl gas was passed through a well cooled and stirred solution of 4-hydroxy-methoxyacetophenone (0.03 mol) and 4-hydroxybenzaldehyde (0.03 mol) in 125 mL of dry ethanol taken in a 250 mL round-bottomed flask for about one hour. Wine red coloured solution was formed to which ice cold water was added. The yellow coloured crystals of (2E)-1-(4-hydroxy-3-methoxyphenyl)-3- (4-hydroxyphenyl)prop-2-en-1-one which got separated was washed with double distilled water and re-crystallized from hot ethanol.

### S3. Refinement

H atoms were positioned geometrically and treated as riding on their parent atoms, with C—H distances of 0.93–0.96 Å, O—H distances of 0.82 Å with *U*_iso_(H) = 1.5 *U*_eq_(C_methyl_) and *U*_iso_(H) = 1.2*U*_eq_(C) for other H atoms. The standard uncertainties on the a and c axes are larger than normal and are indicative of those determined from a poor quality crystal.

### Crystal data

**Table:**

C_16_H_14_O_4_	*D*_x_ = 1.309 Mg m^−^^3^
*M**_r_* = 270.27	Mo *K*α radiation, λ = 0.71073 Å
Orthorhombic, *P**n**a*2_1_	Cell parameters from 2996 reflections
*a* = 7.686 (16) Å	θ = 1.4–28.6°
*b* = 28.346 (7) Å	µ = 0.09 mm^−^^1^
*c* = 6.297 (12) Å	*T* = 293 K
*V* = 1371.9 (5) Å^3^	Block, yellow
*Z* = 4	0.35 × 0.30 × 0.25 mm
*F*(000) = 568	

### Data collection

**Table:**

Bruker Kappa APEXII diffractometer	1928 reflections with *I* > 2σ(*I*)
Radiation source: fine-focus sealed tube	*R*_int_ = 0.055
ω and φ scans	θ_max_ = 28.6°, θ_min_ = 1.4°
Absorption correction: multi-scan (*SADABS*; Bruker, 2008)	*h* = −10→7
*T*_min_ = 0.968, *T*_max_ = 0.977	*k* = −38→19
6436 measured reflections	*l* = −8→8
2996 independent reflections	

### Refinement

**Table:**

Refinement on *F*^2^	Hydrogen site location: inferred from neighbouring sites
Least-squares matrix: full	H-atom parameters constrained
*R*[*F*^2^ > 2σ(*F*^2^)] = 0.077	*w* = 1/[σ^2^(*F*_o_^2^) + (0.1225*P*)^2^ + 0.7988*P*] where *P* = (*F*_o_^2^ + 2*F*_c_^2^)/3
*wR*(*F*^2^) = 0.244	(Δ/σ)_max_ = 0.001
*S* = 1.07	Δρ_max_ = 0.33 e Å^−^^3^
2996 reflections	Δρ_min_ = −0.30 e Å^−^^3^
186 parameters	Absolute structure: Refined as an inversion twin.
1 restraint	Absolute structure parameter: −2 (4)

### Special details

**Table:**

Geometry. All e.s.d.'s (except the e.s.d. in the dihedral angle between two l.s. planes) are estimated using the full covariance matrix. The cell e.s.d.'s are taken into account individually in the estimation of e.s.d.'s in distances, angles and torsion angles; correlations between e.s.d.'s in cell parameters are only used when they are defined by crystal symmetry. An approximate (isotropic) treatment of cell e.s.d.'s is used for estimating e.s.d.'s involving l.s. planes.
Refinement. Refined as a two-component inversion twin (1113 Friedel pairs).

### Fractional atomic coordinates and isotropic or equivalent isotropic displacement parameters (Å^2^)

**Table:**

	*x*	*y*	*z*	*U*_iso_*/*U*_eq_	
O1	−0.3786 (6)	0.30979 (16)	−0.5710 (8)	0.0619 (14)	
O2	−0.1472 (7)	0.59683 (14)	−0.4180 (10)	0.0668 (14)	
H2A	−0.1936	0.6120	−0.5132	0.100*	
O3	−0.0576 (6)	0.16926 (13)	0.0881 (8)	0.0509 (11)	
H3A	−0.0369	0.1764	0.2118	0.076*	
O4	−0.2259 (7)	0.15243 (14)	−0.2582 (7)	0.0554 (12)	
C1	−0.1999 (8)	0.5514 (2)	−0.4288 (11)	0.0489 (14)	
C2	−0.1264 (9)	0.5197 (2)	−0.2908 (11)	0.0532 (16)	
H2	−0.0474	0.5303	−0.1897	0.064*	
C3	−0.1667 (9)	0.4730 (2)	−0.2989 (11)	0.0543 (17)	
H3	−0.1154	0.4522	−0.2031	0.065*	
C4	−0.2855 (8)	0.4557 (2)	−0.4510 (11)	0.0477 (15)	
C5	−0.3587 (9)	0.4886 (2)	−0.5881 (12)	0.0547 (17)	
H5	−0.4368	0.4782	−0.6908	0.066*	
C6	−0.3199 (9)	0.5359 (2)	−0.5777 (11)	0.0552 (17)	
H6	−0.3734	0.5572	−0.6692	0.066*	
C7	−0.3207 (8)	0.4055 (2)	−0.4780 (12)	0.0526 (17)	
H7	−0.3885	0.3972	−0.5946	0.063*	
C8	−0.2672 (8)	0.3700 (2)	−0.3556 (11)	0.0497 (16)	
H8	−0.2055	0.3772	−0.2328	0.060*	
C9	−0.3000 (8)	0.3208 (2)	−0.4035 (11)	0.0478 (14)	
C10	−0.2331 (7)	0.2830 (2)	−0.2685 (11)	0.0400 (13)	
C11	−0.1520 (8)	0.2909 (2)	−0.0730 (10)	0.0458 (15)	
H11	−0.1373	0.3216	−0.0243	0.055*	
C12	−0.0934 (8)	0.2536 (2)	0.0485 (11)	0.0470 (15)	
H12	−0.0376	0.2595	0.1768	0.056*	
C13	−0.1165 (8)	0.2079 (2)	−0.0180 (10)	0.0430 (14)	
C14	−0.2036 (8)	0.1996 (2)	−0.2123 (9)	0.0406 (13)	
C15	−0.2577 (8)	0.2362 (2)	−0.3315 (9)	0.0424 (14)	
H15	−0.3132	0.2302	−0.4599	0.051*	
C16	−0.3006 (10)	0.1410 (2)	−0.4595 (11)	0.0587 (18)	
H16A	−0.3047	0.1073	−0.4757	0.088*	
H16B	−0.2310	0.1543	−0.5708	0.088*	
H16C	−0.4165	0.1535	−0.4673	0.088*	

### Atomic displacement parameters (Å^2^)

**Table:**

	*U*^11^	*U*^22^	*U*^33^	*U*^12^	*U*^13^	*U*^23^
O1	0.064 (3)	0.060 (3)	0.062 (3)	−0.001 (2)	−0.022 (3)	0.010 (2)
O2	0.082 (4)	0.045 (3)	0.072 (3)	0.005 (2)	−0.019 (3)	−0.001 (3)
O3	0.061 (3)	0.046 (2)	0.045 (2)	−0.0050 (19)	−0.012 (2)	0.007 (2)
O4	0.077 (3)	0.040 (2)	0.049 (3)	−0.007 (2)	−0.010 (2)	0.001 (2)
C1	0.049 (3)	0.049 (3)	0.049 (3)	0.009 (3)	−0.001 (3)	0.000 (3)
C2	0.053 (4)	0.055 (4)	0.051 (4)	0.007 (3)	−0.013 (3)	0.002 (3)
C3	0.056 (4)	0.055 (4)	0.051 (4)	0.011 (3)	−0.014 (4)	0.006 (3)
C4	0.038 (3)	0.049 (3)	0.057 (4)	0.004 (2)	−0.002 (3)	0.009 (3)
C5	0.051 (4)	0.056 (4)	0.057 (4)	0.001 (3)	−0.019 (3)	0.008 (3)
C6	0.053 (4)	0.052 (4)	0.060 (4)	0.007 (3)	−0.009 (4)	0.015 (3)
C7	0.042 (4)	0.054 (4)	0.061 (4)	−0.001 (3)	−0.006 (3)	0.008 (3)
C8	0.046 (3)	0.049 (4)	0.054 (4)	−0.002 (3)	−0.005 (3)	0.007 (3)
C9	0.041 (3)	0.048 (4)	0.054 (4)	0.001 (2)	0.002 (3)	0.006 (3)
C10	0.025 (3)	0.049 (3)	0.046 (3)	−0.003 (2)	−0.001 (2)	0.001 (3)
C11	0.042 (3)	0.042 (3)	0.053 (4)	0.001 (2)	−0.004 (3)	−0.003 (3)
C12	0.043 (3)	0.052 (4)	0.046 (4)	−0.006 (2)	0.002 (3)	0.000 (3)
C13	0.038 (3)	0.050 (3)	0.041 (3)	−0.003 (2)	−0.001 (3)	0.004 (3)
C14	0.038 (3)	0.043 (3)	0.041 (3)	−0.005 (2)	0.002 (3)	0.003 (2)
C15	0.033 (3)	0.053 (4)	0.042 (3)	−0.002 (2)	−0.001 (2)	0.002 (2)
C16	0.064 (4)	0.059 (4)	0.053 (4)	−0.012 (3)	−0.008 (4)	−0.004 (3)

### Geometric parameters (Å, º)

**Table:**

O1—C9	1.256 (8)	C7—C8	1.331 (9)
O2—C1	1.351 (7)	C7—H7	0.9300
O2—H2A	0.8200	C8—C9	1.449 (9)
O3—C13	1.361 (7)	C8—H8	0.9300
O3—H3A	0.8200	C9—C10	1.462 (8)
O4—C14	1.378 (7)	C10—C15	1.396 (8)
O4—C16	1.429 (8)	C10—C11	1.398 (9)
C1—C2	1.372 (9)	C11—C12	1.381 (9)
C1—C6	1.387 (9)	C11—H11	0.9300
C2—C3	1.360 (9)	C12—C13	1.371 (8)
C2—H2	0.9300	C12—H12	0.9300
C3—C4	1.412 (9)	C13—C14	1.414 (8)
C3—H3	0.9300	C14—C15	1.348 (8)
C4—C5	1.390 (9)	C15—H15	0.9300
C4—C7	1.459 (8)	C16—H16A	0.9600
C5—C6	1.375 (9)	C16—H16B	0.9600
C5—H5	0.9300	C16—H16C	0.9600
C6—H6	0.9300		
			
C1—O2—H2A	109.5	O1—C9—C8	119.9 (6)
C13—O3—H3A	109.5	O1—C9—C10	118.4 (5)
C14—O4—C16	117.2 (5)	C8—C9—C10	121.6 (6)
O2—C1—C2	118.0 (6)	C15—C10—C11	117.5 (5)
O2—C1—C6	122.4 (6)	C15—C10—C9	118.9 (6)
C2—C1—C6	119.6 (6)	C11—C10—C9	123.5 (5)
C3—C2—C1	121.3 (6)	C12—C11—C10	120.8 (5)
C3—C2—H2	119.3	C12—C11—H11	119.6
C1—C2—H2	119.3	C10—C11—H11	119.6
C2—C3—C4	120.7 (6)	C13—C12—C11	120.7 (6)
C2—C3—H3	119.6	C13—C12—H12	119.6
C4—C3—H3	119.6	C11—C12—H12	119.6
C5—C4—C3	116.7 (6)	O3—C13—C12	124.5 (6)
C5—C4—C7	120.5 (6)	O3—C13—C14	116.6 (5)
C3—C4—C7	122.6 (5)	C12—C13—C14	118.9 (5)
C6—C5—C4	122.5 (6)	C15—C14—O4	126.3 (6)
C6—C5—H5	118.7	C15—C14—C13	119.9 (5)
C4—C5—H5	118.7	O4—C14—C13	113.8 (5)
C5—C6—C1	119.0 (6)	C14—C15—C10	122.1 (6)
C5—C6—H6	120.5	C14—C15—H15	118.9
C1—C6—H6	120.5	C10—C15—H15	118.9
C8—C7—C4	127.7 (7)	O4—C16—H16A	109.5
C8—C7—H7	116.1	O4—C16—H16B	109.5
C4—C7—H7	116.1	H16A—C16—H16B	109.5
C7—C8—C9	123.5 (7)	O4—C16—H16C	109.5
C7—C8—H8	118.2	H16A—C16—H16C	109.5
C9—C8—H8	118.2	H16B—C16—H16C	109.5
			
O2—C1—C2—C3	−176.8 (6)	O1—C9—C10—C11	−176.4 (6)
C6—C1—C2—C3	1.0 (10)	C8—C9—C10—C11	7.9 (9)
C1—C2—C3—C4	0.3 (10)	C15—C10—C11—C12	2.2 (8)
C2—C3—C4—C5	−0.6 (10)	C9—C10—C11—C12	179.2 (6)
C2—C3—C4—C7	174.4 (7)	C10—C11—C12—C13	−1.3 (9)
C3—C4—C5—C6	−0.4 (10)	C11—C12—C13—O3	177.6 (6)
C7—C4—C5—C6	−175.5 (7)	C11—C12—C13—C14	−0.8 (8)
C4—C5—C6—C1	1.7 (10)	C16—O4—C14—C15	5.5 (9)
O2—C1—C6—C5	175.8 (7)	C16—O4—C14—C13	−175.3 (5)
C2—C1—C6—C5	−2.0 (10)	O3—C13—C14—C15	−176.6 (5)
C5—C4—C7—C8	−176.8 (6)	C12—C13—C14—C15	1.9 (8)
C3—C4—C7—C8	8.5 (11)	O3—C13—C14—O4	4.1 (7)
C4—C7—C8—C9	−175.9 (6)	C12—C13—C14—O4	−177.3 (5)
C7—C8—C9—O1	2.0 (10)	O4—C14—C15—C10	178.2 (5)
C7—C8—C9—C10	177.6 (6)	C13—C14—C15—C10	−1.0 (9)
O1—C9—C10—C15	0.5 (9)	C11—C10—C15—C14	−1.1 (8)
C8—C9—C10—C15	−175.2 (5)	C9—C10—C15—C14	−178.2 (6)

### Hydrogen-bond geometry (Å, º)

**Table:**

*D*—H···*A*	*D*—H	H···*A*	*D*···*A*	*D*—H···*A*
O2—H2*A*···O3^i^	0.82	2.59	3.060 (6)	118
O2—H2*A*···O4^i^	0.82	2.02	2.833 (7)	172
O3—H3*A*···O1^ii^	0.82	1.87	2.618 (7)	151

Symmetry codes: (i) −*x*−1/2, *y*+1/2, *z*−1/2; (ii) *x*+1/2, −*y*+1/2, *z*+1.
