# Supplementary figures and images for: Crystal structure of (2E)-1-(4-hy­droxy-3-meth­oxy­phen­yl)-3-(4-hy­droxy­phen­yl)prop-2-en-1-one
Source: Acta Crystallogr Sect E Struct Rep Online. 2014 Oct 15;70(Pt 11):o1158–9. doi: 10.1107/S1600536814021953 (PMC4257319; doi:10.1107/S1600536814021953)

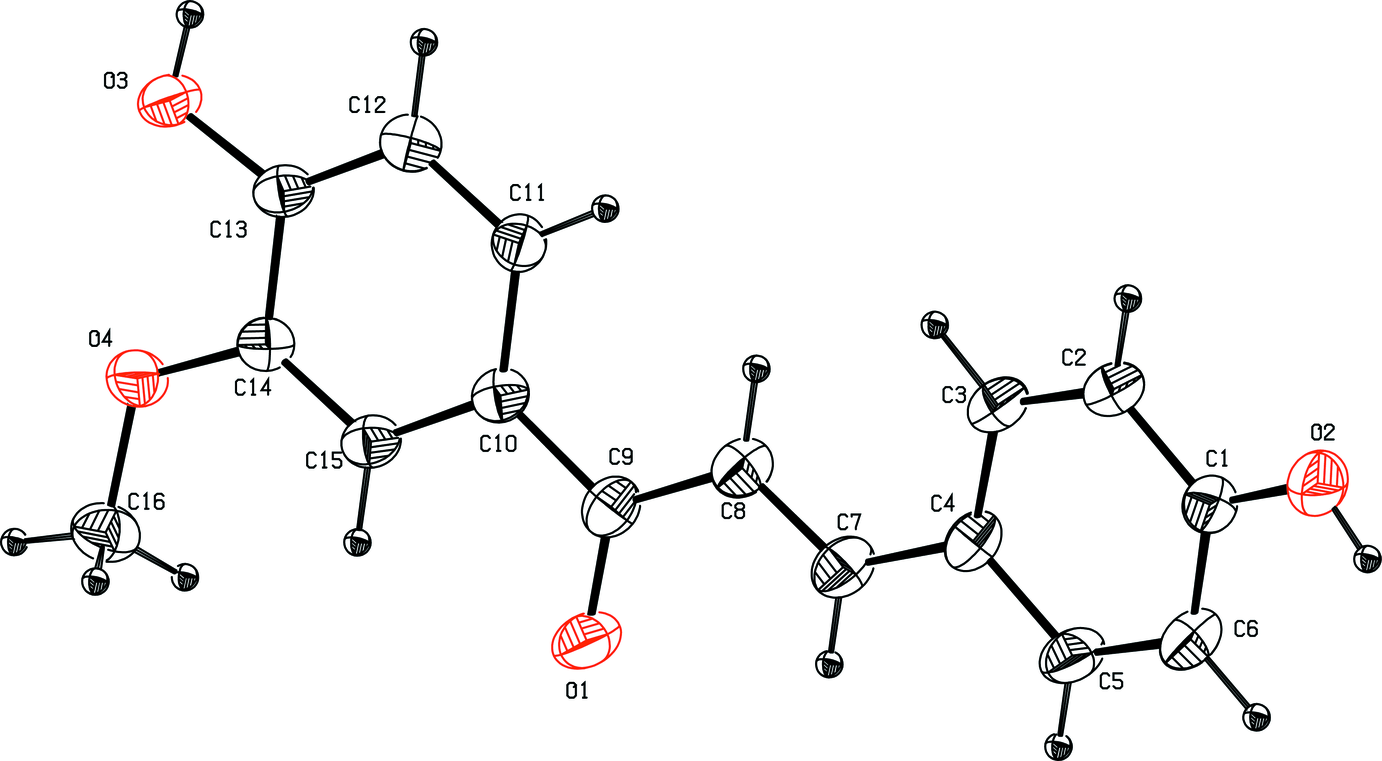

Supplement: Supplementary file 4 [file e-70-o1158-fig1.tif]

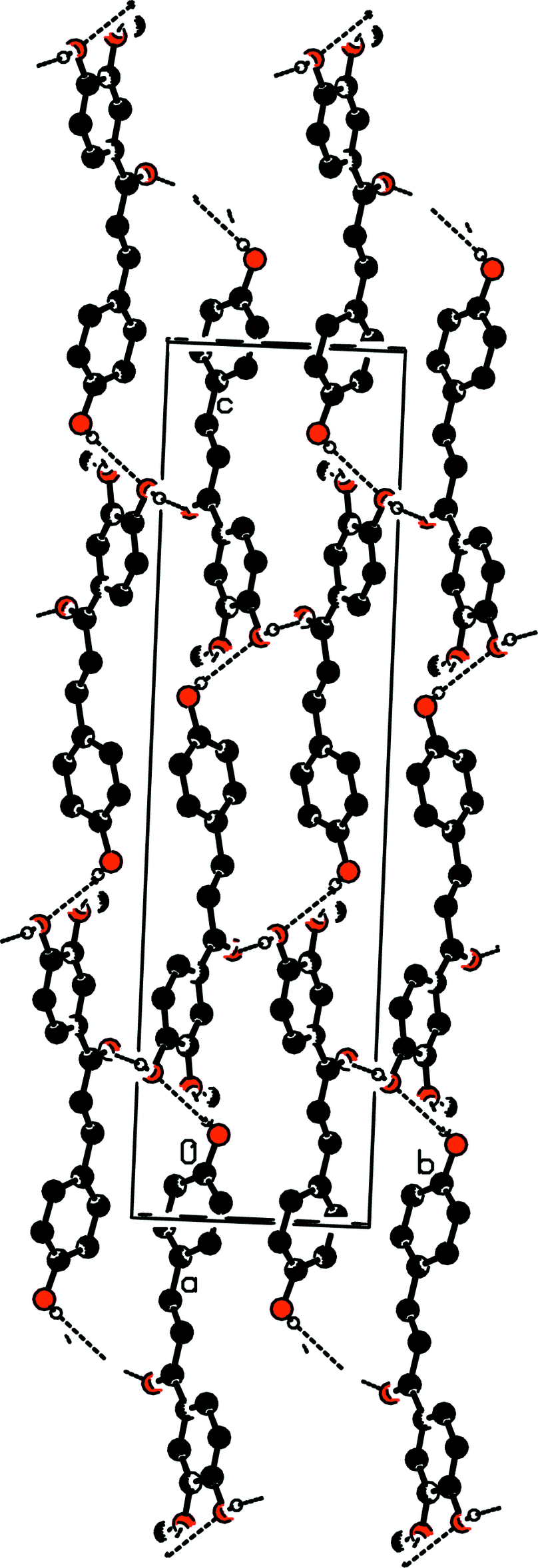

Supplement: Supplementary file 5 [file e-70-o1158-fig2.tif]
